# Diagnostic Yield and Genotype–Phenotype Overlap in Pediatric Autism Spectrum Disorder Patients Using Whole-Exome Sequencing and Phenotype-Driven Variant Interpretation: A Single-Center Cohort Study

**DOI:** 10.3390/children13040444

**Published:** 2026-03-25

**Authors:** Andreya Yaneva, Mariya Levkova, Milena Stoyanova, Mari Hachmeriyan, Lyudmila Angelova, Rouzha Pancheva

**Affiliations:** 1 Department of Medical Genetics, Medical University Varna, Marin Drinov Str. 55, 9000 Varna, Bulgaria; 2 Laboratory of Medical Genetics, University Multiprofile Hospital for Active Treatment “St. Marina”, Hristo Smirnenski Blvd 1, 9000 Varna, Bulgaria; 3 Department of Hygiene and Epidemiology, Faculty of Public Health, Medical University Varna, 9002 Varna, Bulgaria

**Keywords:** autism, whole-exome sequencing, phenotype-genotype correlation, variant interpretation

## Abstract

**Highlights:**

**What are the main findings?**

- Whole-exome sequencing with copy number variation analysis performed in an external diagnostic laboratory identified pathogenic or likely pathogenic variants in 5 of 60 patients, yielded uncertain results in 30, and was negative in 25.
- After clinician-driven reanalysis with full access to clinical data, pathogenic or likely pathogenic variants were identified in 9 patients, a total of 43 variants of unknown significance were detected across 34 patients, and 17 patients had negative results.
- This led to an 80% relative increase in diagnostic yield for pathogenic/likely pathogenic variants.

**What are the implications of the main findings?**

- Clinician-driven, phenotype-based reinterpretation can substantially alter case-level classification, stressing the need for ongoing re-evaluation, segregation studies, and reverse phenotyping to clarify the role of variants of unknown significance over time.
- These findings support comprehensive genomic testing (whole-exome sequencing with copy number variant assessment) in autism spectrum disorder, while highlighting the importance of harmonized interpretation, frameworks between laboratories and structured policies for periodic reanalysis of exome data.

**Abstract:**

**Background/Objectives**: Autism spectrum disorder (ASD) is a clinically and genetically heterogeneous neurodevelopmental condition, and the diagnostic yield of whole-exome sequencing (WES) varies across settings. This single-center study aimed to determine the molecular diagnostic yield of WES in pediatric ASD and to explore genotype–phenotype overlap using a structured, phenotype-driven reanalysis strategy. **Methods:** We enrolled 60 children with syndromic and non-syndromic ASD, who underwent detailed clinical and dysmorphology assessment. WES for single-nucleotide and copy-number variant (CNV) detection was performed in an accredited laboratory, followed by clinician-driven reinterpretation, integrating expanded phenotypic data and ACMG/AMP-based variant classification. Genes were considered if they harbored rare, potentially pathogenic variants and were previously reported or curated in established ASD-associated gene resources. **Results:** The initial external laboratory report identified 5 of 60 patients (8.3%) with a pathogenic (P) or likely pathogenic (LP) variant (positive result), 30 of 60 (50.0%) with a variant of unknown significance (VUS) (inconclusive result), and 25 of 60 (41.7%) with a negative result. Clinician-based variant reinterpretation identified pathogenic or likely pathogenic variants in 9 of 60 patients (15.0%), representing an 80% relative increase in diagnostic yield, as well as 43 VUSs distributed across 34 patients, while 17 patients had no reportable variants (negative result). Overall, reanalysis revealed 11 additional variants of interest (pathogenic, likely pathogenic, or VUS) that had not been reported in the initial assessment. In total, 52 sequence and copy-number variants in 46 genes were detected, most of which were VUSs (83%). **Conclusions:** In this pediatric ASD cohort, WES with phenotype-driven reinterpretation and CNV assessment yielded a clinically positive result in 15% of patients and uncovered additional candidate variants, highlighting both the value and the current interpretative challenge of comprehensive genomic testing in ASD.

## 1. Introduction

Autism spectrum disorders (ASD) is a group of neurodevelopmental conditions, characterized by early-onset impairments in social communication and interaction, accompanied by restricted or repetitive behaviors and interests [1]. The term “spectrum” reflects the wide variability in clinical presentation and severity, ranging from mild functional impairment to profound disability [2]. According to the World Health Organization, ASD affects approximately 1 in 100 children worldwide, highlighting its substantial public health and social impact [1].

ASD typically manifests in early childhood, with most individuals diagnosed before three years of age [3]. Core clinical features include deficits in social interaction and communication, restricted interests and repetitive behaviors, such as hand wringing, and echolalia [1,2,3]. Many individuals with ASD also present with additional neuropsychiatric or somatic comorbidities, such as anxiety, attention-deficit/hyperactivity disorder, epilepsy, sleep disturbances, and gastrointestinal symptoms, which may further influence functional outcomes [3,4]. Despite advances in research, the diagnosis of ASD remains based on clinical evaluation, as no validated molecular or biochemical biomarkers are currently available [2,5].

Over recent decades, the reported prevalence of ASD has increased substantially, rising from approximately 2–4 per 10,000 individuals to an estimated 1 in 160 children, according to the latest reports [4,6,7]. This rise is thought to reflect improved awareness, changes in diagnostic criteria, and broader access to diagnostic services [2,8]. A consistent male predominance has been reported, with ASD diagnosed approximately four times more frequently in males than in females, although delayed or under-recognition in females may partially account for this difference [3,7].

The etiology of ASD is considered multifactorial, involving complex interactions between genetic susceptibility and environmental influences [2,4]. ASD may present as part of a broader syndromic condition or as a non-syndromic disorder (idiopathic ASD) [9]. Syndromic forms are often associated with defined chromosomal abnormalities or monogenic disorders, such as fragile X syndrome and Rett syndrome [2]. In contrast, the etiology of non-syndromic ASD remains incompletely understood, largely due to its extensive genetic heterogeneity [4,7]. The interaction between genetic predisposition and environmental factors is thought to influence both the severity of core autistic features and the presence of associated comorbidities [10].

Over recent years, advances in next-generation sequencing, whole-exome sequencing in particular, have transformed the study of ASD by enabling the systematic identification of rare, high-impact coding variants [10]. WES has therefore become a central tool for uncovering novel ASD-associated variants and elucidating disease mechanisms [10]. However, the substantial variability in analytic pipelines and variant interpretation across centers continues to influence diagnostic yield [11]. Furthermore, although hundreds of ASD susceptibility genes have been identified to date, current evidence suggests that these discoveries represent only a fraction of the underlying genetic architecture, with an estimated 400–1000 genes potentially contributing to ASD susceptibility [12]. De novo and inherited variants in genes such as *CHD8*, *SHANK3*, *SCN2A*, and others involved in synapse formation and neuronal excitability have been repeatedly implicated, alongside chromatin regulators that influence histone modification and gene expression during brain development [13]. At the same time, genome-wide association studies support a highly polygenic architecture in which numerous common variants of individually small effects collectively account for a substantial proportion of ASD heritability, complementing the contribution of rare, high-impact mutations [14].

In this single-center cohort study, we aimed to determine the molecular diagnostic yield of WES in 60 pediatric patients with syndromic and non-syndromic ASD and to investigate genotype–phenotype overlap using a structured, phenotype-driven variant interpretation approach. Additionally, we sought to present data from Bulgarian patients with ASD, as Bulgaria remains underrepresented in the current genomic literature.

## 2. Materials and Methods

### 2.1. Study Cohort

Sixty pediatric patients with ASD, including childhood autism and pervasive developmental disorder, all diagnosed by a child psychiatrist according to the Diagnostic and Statistical Manual of Mental Disorders, 5th Edition (DSM-5) criteria, were enrolled. 

Participants were recruited from children, hospitalized at St. Marina University Hospital, Varna, Bulgaria, for evaluation of ASD across different clinical departments, including pediatrics unit and child psychiatry. Recruitment was conducted using both retrospective and prospective approaches between January 2024 and October 2025. All participants underwent comprehensive clinical evaluation, including medical genetics assessment with evaluation for hereditary disorders, congenital anomalies, and dysmorphic features, prior to inclusion in the study.

Inclusion criteria were: (i) age < 18 years and (ii) a confirmed clinical diagnosis of ASD by a child psychiatrist. Exclusion criteria were: (i) age ≥ 18 years; (ii) alternative primary neurodevelopmental diagnoses (e.g., attention-deficit/hyperactivity disorder, attention-deficit disorder, or isolated developmental speech/language disorder) in the absence of a formal ASD diagnosis; and (iii) prior clinical WES performed before study enrollment.

### 2.2. Clinical Evaluation and Phenotyping

Systematic phenotyping was performed for all participants during in-person consultations with a specialist in medical genetics, as detailed clinical characterization was deemed essential to optimize WES interpretation. For each patient, available medical documentation was reviewed, including discharge summaries from previous hospitalizations and results of prior investigations such as magnetic resonance imaging (MRI) and ultrasonography, when available, to identify structural anomalies of the central nervous system or internal organs.

The standardized clinical assessment included measurement of height, weight, and head circumference; documentation of prenatal and perinatal history; and a detailed developmental history from birth, with particular emphasis on motor milestones, language acquisition, and behavioral phenotype. A three-generation pedigree was obtained for all families. Human Phenotype Ontology (HPO) terms were applied to standardize phenotypic descriptions across the cohort. With written informed consent, standardized facial photographs (frontal and profile views) were acquired and uploaded to the Face2Gene [15,16] platform to support structured phenotypic documentation and to aid in the recognition of possible syndromic patterns.

### 2.3. DNA Extraction and Preliminary Testing

Genomic DNA was obtained from peripheral blood leukocytes, and DNA extraction was performed using standard laboratory protocols to ensure sufficient quantity and quality for downstream analysis. All DNA samples were subsequently sent to an accredited external laboratory for WES.

As part of the initial genetic evaluation, all patients underwent screening for Fragile X syndrome using methylation-sensitive multiplex ligation-dependent probe amplification (MS-MLPA) (SALSA MS-MLPA Probemix ME029, FMR1-AFF2, MRC-Holland, Amsterdam, The Netherlands). Results were negative in all cases.

### 2.4. Whole-Exome Sequencing

Whole-exome sequencing was performed in an ISO-certified external laboratory using a standardized clinical protocol. Libraries were prepared with a clinical exome capture panel and sequenced on an Illumina NovaSeq platform (NovaSeq 6000, Illumina, Inc., San Diego, CA, USA) achieving high coverage (mean depth > 100× with >99% of targeted bases at ≥20×), sufficient for comprehensive detection of single-nucleotide variants (SNVs) and small insertions/deletions (INDELs).

### 2.5. Bioinformatic Processing

Sequencing data was processed using the laboratory’s validated clinical pipeline, following current best-practice guidelines for read alignment, variant calling, and quality control. The workflow included detection of SNVs, INDELs and CNVs, as well as interrogation of mitochondrial variants, repeat expansions, mobile element insertions, and regions of homozygosity, with variant annotation performed using a current version of Variant Effect Predictor (VEP) [17].

### 2.6. Variant Interpretation and Clinician-Driven Reanalysis

Following receipt of the raw sequencing data, all variant calling format (VCF) files were uploaded to the Franklin platform (Genoox, Tel Aviv, Israel) [18] for clinician-driven variant interpretation. This analysis incorporated expanded phenotypic information derived from the comprehensive clinical evaluation, including detailed dysmorphology assessment, neuroimaging findings, comorbidities, and pedigree data.

The variant interpretation workflow consisted of several steps. First, candidate variants highlighted on the Franklin “workbench” as most likely to explain the phenotype were reviewed using clinical judgment. Heterozygous variants in genes associated with autosomal recessive conditions were excluded, unless a second pathogenic or likely pathogenic allele was identified. In parallel, variants were systematically evaluated against an ASD-focused gene panel derived from the Australian PanelApp resource (Supplementary Table S1); within this panel, variants classified as pathogenic, likely pathogenic, or variants of uncertain significance with a tendency toward pathogenicity were added to the workbench for detailed review. For secondary (incidental) findings, genes published in ClinVar [19] for pathogenic and likely pathogenic variants were screened, using a gene list curated according to American College of Medical Genetics and Genomics (ACMG) recommendations for reporting secondary findings [20]. Finally, additional analyses were performed to identify homozygous variants of potential relevance.

Variants were prioritized based on multiple lines of evidence, including: (i) technical quality metrics (read depth, allelic balance, mapping quality) to exclude sequencing artifacts; (ii) inheritance pattern; (iii) predicted molecular consequence (protein-truncating, canonical splice-site, or missense variants in constrained genes); (iv) population allele frequency in gnomAD [21], with rare variants retained using thresholds informed by disease prevalence and American College of Medical Genetics and Genomics and the Association for Molecular Pathology (ACMG/AMP)-based recommendations; and (v) gene–disease validity curated in resources such as ClinGen [22], Online Mendelian Inheritance in Man (OMIM) [23], and GenCC [24], with preference for genes classified as having definitive, strong, or moderate evidence for association with neurodevelopmental disorders (NDD) or ASD. In silico pathogenicity predictions (e.g., REVEL, CADD, AlphaMissense, SpliceAI) were used as supportive evidence. Final inclusion of a variant in the clinical report required classification as pathogenic, likely pathogenic or VUS, according to ACMG/AMP or adapted frameworks, together with a high degree of concordance between the gene’s known or proposed phenotype and the individual’s clinical presentation, with a focus on neurodevelopmental and ASD features.

Copy-number variant analysis was initially performed by the external laboratory using its validated clinical exome pipeline, which includes read-depth-based CNV calling. As an additional quality-control step, candidate regions were reviewed in Integrative Genomics Viewer (IGV, Broad Institute, Cambridge, MA, USA; https://igv.org/), with the display scale set from −2 to +2 and a window size of 400, and each chromosome systematically inspected for consistent decreases or increases in depth across exons relative to neighboring regions. CNVs of potential interest were further evaluated using public databases such as the University of California, Santa Cruz (UCSC) Genome Browser [25], DECIPHER [26], and CNVHub [27] to assess genomic size, gene content, and population frequencies. In the present study, non-diagnostic CNVs were not validated by orthogonal methods, and the single clearly pathogenic USP7 deletion had been confirmed by the reporting laboratory as part of their routine clinical workflow.

All variants were classified according to ACMG/AMP guidelines and evaluated for secondary findings [20,28]. Variants of uncertain significance were further stratified into three categories—low, medium, and high phenotype overlap—based on the degree of concordance between the patient’s clinical presentation and the reported gene–disease association.

### 2.7. Ethics

Written informed consent was obtained from the parents or legal guardians of all participants, as all enrolled individuals were minors. Consent covered participation in the study, processing of biological material, and handling of anonymized personal data, including clinical records and facial photographs.

The study protocol, consent forms, and data processing procedures were reviewed and approved by the Institutional Ethics Committee of Medical University Varna. All study procedures were conducted in accordance with the Declaration of Helsinki and relevant national regulations on biomedical research involving human subjects.

### 2.8. Statistical Analysis

Descriptive statistics were used to summarize clinical characteristics, variant categories, and diagnostic outcomes in the cohort. Categorical variables (e.g., presence of developmental delay, intellectual disability, dysmorphic features, variant classification, and WES result category) are reported as counts and percentages. Continuous variables (e.g., age at inclusion) are presented as mean and standard deviation. Given the exploratory design and relatively small sample size, no formal hypothesis testing or multivariable modeling was performed; analyses were limited to descriptive comparisons of proportions between groups (e.g., initial laboratory report versus clinician-driven reinterpretation).

## 3. Results

### 3.1. Phenotypic Features

The cohort included 54 males and 6 females, all between the ages of 3 and 18 years at the time of inclusion. The mean age at inclusion was 7.9 (SD ± 3.6 years).

The cohort was clinically heterogeneous with respect to comorbidities. Additional neurodevelopmental or syndromic features—including dysmorphic features, intellectual disability or developmental delay, epilepsy, and/or congenital malformations—were present in all of the patients in variable combinations, whereas only 3 patients presented with ASD and age-appropriate cognitive functioning (high-functioning ASD).

Developmental delay was present in the vast majority of participants (96.6%), followed by intellectual disability seen in 70%. Dysmorphic features were observed in 61.6%, while additional congenital or other anomalies were documented in 46.6%. Gastrointestinal comorbidities, such as lactose or gluten intolerance, were reported in 25%. Epilepsy was present in 16.6%, and proven structural brain anomalies on MRI were identified in 10.0% of patients (Table 1). In this study, we defined syndromic ASD as ASD accompanied by additional clinical features suggestive of an underlying genetic syndrome, such as characteristic dysmorphic features, congenital anomalies, abnormal growth parameters, and/or major neurological comorbidities. In contrast, non-syndromic ASD referred to ASD without major dysmorphology or major congenital anomalies after detailed clinical assessment [29]. Overall, based on the combined presence of additional dysmorphic features, congenital anomalies, and/or other neurodevelopmental comorbidities, 36 of 60 patients (60%) were classified as having syndromic ASD, whereas 24 of 60 (40%) were considered non-syndromic ASD. A detailed distribution of key clinical characteristics (including dysmorphic vs. non-dysmorphic appearance, and presence or absence of congenital anomalies) is provided in Supplementary Table S2.

When focusing strictly on core ASD-related features, the most prevalent findings in our cohort were stereotypic behaviors, speech delay, and poor eye contact (Table 2).

### 3.2. Variant Interpretation

In the initial laboratory report, 5 of 60 patients (8.3%) were found to harbor a pathogenic or likely pathogenic variant, resulting in a positive diagnosis; 30 (50.0%) received an uncertain diagnosis with a reported VUS, and 25 (41.7%) had negative results.

**Figure 1:**
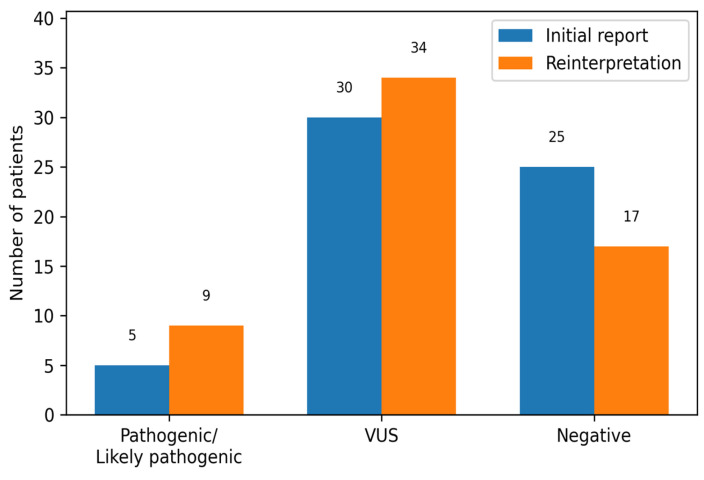
Whole-exome sequencing results before and after clinician-driven reinterpretation in the pediatric ASD cohort.

After uploading the VCF files to Franklin and incorporating detailed clinical information, 9 of 60 patients (15.0%) were found to carry a pathogenic or likely pathogenic variant (meaning a positive results), 34 patients (56.7%) fell into the inconclusive diagnosis category, each harboring at least one reported VUS, and 17 (28.3%) had no variants considered worthy of reporting (negative result). In total, across the cohort, 43/60 patients (71.67%) carried at least one variant of interest (pathogenic, likely pathogenic, or VUS) (Table 3), compared to the initial laboratory analysis which returned 35/60 (58.33%) cases in which a variant of interest was reported (Figure 1).

From a molecular point of view, in our reanalysis, we identified a total of 52 variants, including 43 VUS, 6 likely pathogenic, and 3 pathogenic variants. The discrepancy between the number of diagnoses and number of variants found, is explained by the fact that a subset of patients (*n* = 6) harbored more than one VUS simultaneously. Additionally, 11 variants were not reported in the original laboratory assessment.

The 52 variants affected 46 unique genes, indicating that some genes (*n* = 6) were repeated twice in our cohort (*TRRAP*, *ISN1*, *MACF1*, *ADNP*, *NIPBL*, *TAOK2*). The detected variants comprised 37 missense (71.15%), 7 splice-site (13.46%), 4 frameshift (7.69%), 2 nonsense (3.85%), 1 in-frame indel (1.9%), and 1 CNV (1.9%). When determining the phenotype–genotype concordance for these variants, it was judged to be high in 54.7%, moderate in 34.0%, and low in 7.5% of cases.

There was only one pathogenic CNV—a 69.3 kb deletion affecting exons 1–31 of *USP7* (16p13.2)—which was classified as diagnostic for Hao–Fountain syndrome, whereas the remaining CNVs were considered non-diagnostic.

The pathogenic/likely pathogenic variants were in the following genes: *SLC9A9* (autism spectrum disorder), *WAC* (DeSanto–Shinawi syndrome), *NRXN2* (autism spectrum disorder), *WDYF3* (WDFY3-related primary microcephaly or macrocephaly with developmental delay), *NSD1* (Sotos syndrome), a deletion in *USP7* (Hao–Fountain syndrome), *NIPBL* (Cornelia de Lange syndrome), *ATP1A3* (developmental and epileptic encephalopathy 99) and *NARS* (Neurodevelopmental disorder with microcephaly, impaired language, epilepsy, and gait abnormalities (Autosomal Dominant)) (Table 4).

Across the VUS set, most variants were supported by PM2 (pathogenic, moderate evidence: absent/rare in population databases) alone or PM2 plus a single computational criterion, with 29/43 (67.4%) carrying PM2 as the only pathogenic criterion (often with PP3 [pathogenic, supporting: multiple computational lines in favor] or BP4 [benign, supporting: multiple computational lines against] at supporting or moderate strength) and no strong or very strong evidence. A smaller subset, 7/43 (16.3%), harbored a PVS1_Moderate (predicted loss-of-function variant in a gene where loss of function (LOF) is a known mechanism) or PM1 (pathogenic, moderate: located in a critical/functional domain) code together with PM2, but still did not reach the evidence threshold for likely pathogenic due to lack of de novo, segregation, or robust functional data. In 5/43 variants (11.6%), benign evidence (BS1 [benign, strong: allele frequency too high], BS2 [benign, strong: observed in healthy individuals], or BP4) partially counterbalanced pathogenic criteria, yielding mixed profiles that resolved as VUS under standard combining rules. Only 2/43 VUSs (4.7%) approached a “VUS-leaning pathogenic” profile (multiple moderate plus supporting pathogenic criteria), but remained below the level required for reclassification in the absence of stronger phenotype-specific or segregation evidence (Supplementary Table S3).

## 4. Discussion

This single-center whole-exome sequencing study of 60 pediatric patients with autism spectrum disorder and associated neurodevelopmental features demonstrates a modest yet clinically meaningful diagnostic yield, together with a substantial burden of variants of uncertain significance.

Chromosomal microarray analysis (CMA) was not performed as a separate first-line test, because recent guidelines and emerging evidence increasingly support exome or genome sequencing as a first-tier (or early-tier) approach in children with unexplained developmental delay, intellectual disability, and/or ASD, and our strategy followed these recommendations [30].

Using the algorithm described above, a definitive molecular diagnosis was established in 9 of 60 patients (15%). These values place our detection rate at the lower end of the range reported for ASD within broader neurodevelopmental cohorts, but closely match several ASD-focused exome studies that also report yields around 8–16% when prior chromosomal microarray or targeted testing has been performed [31,32,33,34].

A key feature of our approach is that reanalysis was primarily clinician-driven. This workflow mirrors reports showing that re-evaluation “by clinical geneticists involved in the medical care of the individuals tested” improves diagnostic yield beyond that achieved by bioinformatic reannotation alone [35]. While laboratory pipelines must rely on predefined filters and often limited clinical descriptions, clinicians can refine phenotype terms, recognize subtle dysmorphic features or neurological signs, and contextualize behavioral manifestations in ways that directly influence genotype–phenotype matching [36]. Furthermore, not only do they know the child’s developmental history and comorbidities but also follow the evolution of the phenotype over time [36]. This close link between bedside observation and bioinformatic reanalysis likely explains a substantial part of the increased yield observed in our cohort. It also implies that centers wishing to optimize the impact of exome sequencing in ASD should build workflows in which clinicians routinely revisit and refine both the phenotypic and molecular data [36].

When comparing the diagnostic yield between the original laboratory report and our clinician-driven reinterpretation, the proportion of positive cases increased from 8.3% to 15.0%, while the share of patients with an uncertain diagnosis rose from 50.0% to 56.7% and those classified as clearly negative decreased from 41.7% to 28.3%. This shift indicates that phenotype-focused reanalysis not only uncovered additional pathogenic or likely pathogenic variants, but also moved some previously negative cases into the uncertain category, thereby reducing the number of children considered definitively negative and expanding the group for whom further investigation is warranted. Overall, the 80% relative increase in definitive diagnoses and the identification of 11 additional variants highlight the added value of integrating detailed, patient-specific clinical information into variant interpretation and support viewing exome data as a dynamic resource that benefits from periodic clinician-driven reinterpretation rather than a one-time static test [37].

The spectrum of variants detected in our study is consistent with previous work in ASD and neurodevelopmental disorders [31,32,33,34]. We identified 51 SNVs and 1 CNV across 46 unique genes, the majority being missense variants (42/52, 71.15%) (Table 3). This predominance of rare missense changes with a smaller proportion of truncating variants mirrors the distributions reported by other WES cohorts in ASD, where many candidate variants localized to genes with known or suspected roles in brain development, synaptic function, or chromatin regulation [38]. Additionally, this pattern is consistent with the current understanding that ASD arises from a broad and overlapping set of genes with variable penetrance and expressivity, and that many rare, potentially damaging variants remain difficult to classify confidently [39].

The positive diagnoses in this cohort involved genes with well-established roles in syndromic neurodevelopmental disorders frequently associated with ASD traits, including *WAC* (c.374_378+1del, DeSanto–Shinawi syndrome), *NSD1* (c.4302+1G>T, Sotos syndrome), *NIPBL* (c.5689_5691del, Cornelia de Lange syndrome), *USP7* (Deletion of exons 1–31, Hao–Fountain syndrome), *SLC9A9* (c.374_378+1del, autism spectrum disorder) and others (Table 3).

All of these genes are compatible in a varying degree with the combinations of developmental delay, intellectual disability, dysmorphic features, and behavioral abnormalities observed in our patients. This pattern is in line with other exome-based ASD studies, where a substantial fraction of “solved” cases represent recognizable syndromic conditions in which ASD constitutes part of a broader phenotype [40]. Our findings therefore support the notion that, particularly in children with dysmorphic features, congenital anomalies, or additional neurological signs, WES can reveal classic neurodevelopmental syndromes rather than isolated idiopathic ASD [33,41,42].

Recurrently affected genes in our cohort—MACF1, TAOK2, ADNP, NIPBL, ITSN1 and TRRAP, each of them seen twice in our small cohort of 60 patients—further strengthen the emerging or established links between these genes and ASD. Several of them are implicated in neuronal development, axonal guidance, synaptic function, or chromatin remodeling, and have appeared as candidate or confirmed genes in other ASD and NDDs datasets [43,44,45].

Copy-number analysis yielded one clearly pathogenic CNV—a 69.3 kb deletion encompassing exons 1–31 of USP7—which was classified as diagnostic for Hao–Fountain syndrome and is comparable in size and genomic location to USP7 deletions reported in association with ASD, intellectual disability, and seizures [46]. The phenotypic overlap in this case was considerable, keeping in mind the overlap of the brain anomalies described in the syndrome (OMIM, 616863) and the ones found in our patient—ventriculomegaly decreased white matter, leukomalacia, ASD and dysmorphic features [46].

Despite this improvement, however, the majority of children did not achieve a definitive molecular diagnosis, underscoring the significant genetic heterogeneity of ASD [47]. After reanalysis, 71.7% of patients in our cohort carried at least one variant of interest (pathogenic, likely pathogenic, or VUS), yet only 15.0% had a clearly diagnostic variant, reflecting a large reservoir of uncertain findings.

The large burden of VUS in our cohort—43 variants in 34 patients—illustrates both the power and limitations of current exome-based diagnostics in ASD [48,49]. On one hand, these variants cannot yet be used to guide firm clinical decisions under existing ACMG/AMP criteria; on the other hand, they likely contain a substantial fraction of future diagnoses as gene–disease associations and functional data continue to accumulate [48]. The fact that six patients harbored two VUS simultaneously raises the possibility of oligogenic contributions or blended phenotypes, which are inherently challenging to resolve with current classification frameworks [50].

Within this landscape, our next crucial step is systematic reverse phenotyping, particularly for children carrying VUS in genes associated with syndromic neurodevelopmental disorders. Reverse phenotyping starts from the genotype and prompts a targeted re-examination for clinical features that may have been subtle, age-dependent, or initially overlooked [49,51]. By deliberately searching for signs known to be associated with a given gene, clinicians can clarify the presumed genotype–phenotype relationship and, in some cases, support reclassification of a VUS toward likely pathogenic [52]. In ASD, where phenotypes are often broad and overlapping, structured reverse phenotyping protocols with standardized checklists and longitudinal follow-up are likely to be particularly useful for resolving ambiguous findings [53].

Family-based segregation analysis represents another essential strategy to refine the interpretation of the many VUS uncovered in our cohort. Determining whether a variant arose de novo, segregates with disease in affected relatives, or is carried by unaffected family members provides important evidence for or against pathogenicity [54]. Implementing systematic parental and, when possible, sibling testing for prioritized VUS in our cohort will therefore be critical for reclassifying a subset of these variants and may further increase the diagnostic yield beyond the 15.0% achieved with clinician-based reanalysis alone [55,56].

In the present cohort, parental samples were not available at the moment of writing this manuscript; however, we explored the theoretical impact of segregation data on our VUS set. By simulating a best-case de novo scenario in which segregation analysis would support the variant (i.e., activating the corresponding segregation criterion in the Franklin by Genoox), we found that under a confirmed de novo (PS2) simulation, and after removing variants with conflicting benign evidence (BS2/BP4) 28/43 (65.1%) of VUS would reclassify to Likely Pathogenic, under standard ACMG/AMP combining rules, provided there was no strong phenotype contradiction.

Large neurodevelopmental disorder studies consistently show that yields are higher in children with global developmental delay or intellectual disability, multiple congenital anomalies, or clearly syndromic presentations than in those with isolated ASD [33]. The fact that our detection is at 15% despite a largely syndromic phenotype mix (56,67% of the patients had a dysmorphic feature) suggests that other factors—including sample size, limited trio data, technical constraints of WES, and conservative classification—substantially influenced the final yield. This observation highlights both the potential and the limitations of current exome-based diagnostics in ASD: even in a clinically complex cohort, many cases remain unresolved, yet the presence of extensive comorbidity and dysmorphism supports the likelihood that further clinically relevant variants remain hidden among the VUS.

From a practical perspective, WES with CNV analysis remains a relatively expensive diagnostic approach and is not yet universally reimbursed in Bulgaria. In our center, this strategy is therefore preferred for patients with more complex or syndromic presentations, often after more basic investigations have been completed, i.e., cytogenetic analysis or MLPA. Although upfront costs are higher than for sequential single-gene or small-panel testing, published data in neurodevelopmental disorders suggest that exome-based approaches can be cost-effective by shortening the diagnostic odyssey and reducing repeated, low-yield investigations [57]. Our findings support the view that broader implementation of exome-based diagnostics in ASD could be feasible if coupled with clear clinical selection criteria and appropriate health-system funding mechanisms.

For this reason the following limitations of this study should be considered when interpreting the results. First, the cohort size (60 patients) restricts statistical power to detect subtle genotype–phenotype associations and limits the ability to evaluate recurrence beyond the small number of genes (*MACF1*, *TAOK2*, *ITSN1*, *ADNP*, *TRRAP*, *NIPBL*) affecting two individuals each. Second, the lack of systematic trio analysis for all patients substantially reduces the ability to identify and confidently interpret de novo variants, which are a major contributor to ASD risk. Third, WES has inherent technical limitations: non-coding regulatory variants, deep intronic changes, complex structural variants, low-level mosaicism, and mitochondrial variants may be missed, and exonic coverage is not uniformly complete across all genes [58]. The true proportion of genetically explained cases is therefore likely higher than our observed diagnostic rate. Finally, and central to the point about differences between laboratories, variant interpretation depended on our local implementation of ACMG/AMP criteria, on the databases and literature available at the time of analysis, and on clinical judgment regarding phenotype–genotype fit. Comparative studies have shown considerable inter-laboratory variability in variant classification, including non-trivial rates of disagreement in P/LP versus VUS labels for the same variant [59]. Consequently, some of the variants classified as VUS in this study might be considered LP in other centers or in future re-analyses as additional evidence becomes available, while others currently labeled LP might be downgraded as the field’s understanding evolves.

## 5. Conclusions

Our single-center experience supports three main conclusions. First, WES with integrated CNV analysis achieved a definitive molecular diagnosis in 15% of pediatric ASD cases, mostly identifying syndromic neurodevelopmental disorders with autistic features. Second, the high rate of rare VUSs highlights the need for careful clinician-led phenotype–genotype overlap in variant interpretation and clinical decision-making. Third, systematic segregation studies, reverse phenotyping, and harmonized interpretative frameworks are essential to transform today’s VUSs into tomorrow’s actionable diagnoses. Overall, these findings support an iterative, clinician-centered diagnostic model—where reanalysis, reverse phenotyping, and family testing are integrated over time to fully realize the diagnostic potential of genomic data. For pediatric practice, structured reanalysis workflows and close clinician–laboratory collaboration may yield greater diagnostic gains than expanding sequencing volume alone.

## Figures and Tables

**Table 1:** The table describes the number of patients presenting with a specific phenotype.

Phenotype	Number of Patients
Developmental delay	58
Intellectual deficit	42
Facial dysmorphism	37
Other congenital anomalies	28
Lactose/Gluten intolerance	15
Seizures	10
Brain anomalies on MRI	6

**Table 2:** Subcategorization of ASD-related phenotypes, seen in our cohort, *n* = 60.

Autism-Related Phenotype	N =	
Stereotypic behavior	38	63.33%
Speech delay	37	61.67%
Poor eye contact	26	43.33%
Maladaptive behaviour	14	23.33%
Hyperactivity	12	20.00%
Attention deficit	12	20.00%
Toe walking	10	16.67%
Autoagression	9	15.00%
Delayed motor development	3	5.00%

**Table 3:** Summary of whole-exome sequencing (WES) findings and variant characteristics in the study cohort (N = 60) after clinician-based reanalysis.

WES Findings	N =	
Diagnosis	60	
Positive	9	15%
Uncertain	34	56.67%
Negative	17	28.33%
Variants found	52	
Number of genes affected	46	
Genes, affected >1	6	
Variant classification		
VUS	43	82.69%
LP	6	11.54%
P	3	5.77%
Variant effect		
Missense	37	70.59%
Splice related	7	7.84%
Frameshift	4	13.73%
Nonsense	2	3.92%
Non frameshift	1	1.96%
CNV	1	1.96%

**Table 4:** Genomic description of the 9 P/LP variants of patients (No: 8, 12, 32, 37, 38, 40, 42, 58, 59). All the described variants are heterozygous. ACMG/AMP variant interpretation criteria were applied as follows: PVS1 (pathogenic, very strong) for predicted loss-of-function variants (e.g., nonsense, frameshift, canonical splice-site) in genes where loss of function is an established disease mechanism; PM2 (pathogenic, moderate) for variants absent or extremely rare in population databases; PM1 (pathogenic, moderate) for variants located in a mutational hot spot or a well-established functional domain without benign variation; PM4_Supporting (pathogenic criterion 4, applied at supporting strength) for protein length-altering variants such as in-frame insertions/deletions or stop-loss variants; PS3 (pathogenic, strong) for well-established functional studies supportive of a damaging effect on the gene or protein; PP2 (pathogenic, supporting) for missense variants in genes where missense change is a common disease mechanism and benign missense variation is limited; PP3_Moderate (computational evidence, applied at moderate strength) for multiple in silico predictions supporting a deleterious effect; and PP5_Moderate or PP5_Very_Strong (pathogenic, supporting criterion 5 applied at moderate or very strong strength) for pathogenic assertions from reputable clinical or expert sources, with the strength adjusted according to the robustness and concordance of the external evidence. HGVS—Human Genome Variation Society; HGVSc—HGVS coding DNA sequence nomenclature; HGVSp—HGVS protein sequence nomenclature.

Pt No.	Gene	HGVSc	HGVSp	Effect	ACMG Criteria	Associated Condition
8	*SLC9A9*	c.374_378+1del	p.Glu125fs	Splice donor disruption	PVS1 PM2	Autism spectrum disorder, OMIM 608396
12	*WAC*	c.252_253del	p.His84GlnfsTer3	Frameshift	PVS1 PM2 PP5_Moderate	DeSanto–Shinawi syndrome, OMIM 615049
32	*NRXN2*	c.148_157del	--	Frameshift	PVS1 PM2	Autism spectrum disorder
37	*WDFY*3	c.8902-2A>G	--	Splice acceptor, intron change	PVS1 PM2	WDFY3-related primary microcephaly or macrocephaly with developmental delay
38	*NSD1*	c.4302+1G>T	--	Canonical splice donor (+1)	PVS1 PM2	Sotos syndrome, OMIM 117550
40	*USP7*	Deletion of exons 1–31	--	CNV—Deletion (~69.3 kb)		Hao–Fountain syndrome, OMIM 616863
42	*NIPBL*	c.5689_5691del	p.Asn1897del	Non frame shift deletion	PS3 PM2 PM4_Supporting PP5_Very_Strong	Cornelia de Lange, OMIM 300590
58	ATP1A3	c.1097A>G	p.Asp366Gly	Missense	PM1 PM2 PP2 PP3_Moderate	Developmental and epileptic encephalopathy 99
59	NARS	c.1067A>C	p.Asp356Ala	Missense	PM2 PP5	Neurodevelopmental disorder with microcephaly, impaired language, epilepsy, and gait abnormalities (Autosomal Dominant)

## Data Availability

The original contributions presented in this study are included in the article/Supplementary Material. Further inquiries can be directed to the corresponding author.

## Supplementary Materials

The following supporting information can be downloaded at: https://www.mdpi.com/article/10.3390/children13040444/s1, Table S1: Autism gene panel, v.0.243, taken from https://panelapp-aus.org/panels/, accessed on 30 January 2026; found in Supplementary Materials; Table S2: Distribution of core clinical and dysmorphic features, found in Supplementary Materials; Table S3: Detailed description of the found variants of unknown significance, following ACMG criteria, found in Supplementary Materials.

## Abbreviations

The following abbreviations are used in this manuscript:

ACMG	American College of Medical Genetics and Genomics
ACMG/AMP	American College of Medical Genetics and Genomics and the Association for Molecular Pathology
ASD	Autism spectrum disorder(s)
BP	Benign Supporting (ACMG evidence code prefix)
BS	Benign Strong (ACMG evidence code prefix)
CMA	Chromosomal microarray analysis
CNV	Copy-number variant
DNA	Deoxyribonucleic acid
DSM-5	Diagnostic and Statistical Manual of Mental Disorders, 5th Edition
HGVS	Human Genome Variation Society
HGVSc	HGVS coding DNA sequence nomenclature
HGVSp	HGVS protein sequence nomenclature
HPO	Human Phenotype Ontology
IGV	Integrative Genomics Viewer
INDEL	Insertion/deletion
ISO	International Organization for Standardization
LOF	Loss of function
LP	Likely pathogenic
MRI	Magnetic resonance imaging
NDD	Neurodevelopmental disorder(s)
OMIM	Online Mendelian Inheritance in Man
P	Pathogenic
PM	Pathogenic Moderate (ACMG evidence code prefix)
PS	Pathogenic Strong (ACMG evidence code prefix)
PVS	Pathogenic Very Strong (ACMG evidence code prefix)
SNV	Single-nucleotide variant
VCF	Variant call(ing) format
VEP	Variant Effect Predictor
VUS	Variant of unknown significance
WES	Whole-exome sequencing
